# Individual and organizational factors associated with public health workforce competencies to advance health equity

**DOI:** 10.1371/journal.pgph.0004068

**Published:** 2025-01-09

**Authors:** Paula M. Kett, Shahida Shahrir, Betty Bekemeier, Kay Schaffer, Danielle J. Zemmel, Davis G. Patterson

**Affiliations:** 1 Center for Health Workforce Studies, University of Washington School of Medicine, Seattle, Washington, United States of America; 2 Department of Child, Family and Population Health, University of Washington School of Nursing, Seattle, Washington, United States of America; 3 De Beaumont Foundation, Bethesda, Maryland, United States of America; 4 Region V Public Health Training Center, University of Michigan School of Public Health, Ann Arbor, Michigan, United States of America; University of Washington Department of Global Health, UNITED STATES OF AMERICA

## Abstract

Little is known about how to develop public health workforce capacity for health equity work. We explored associations of individual and organizational characteristics of local public health departments (LHDs) with competencies essential for advancing health equity. Data included responses of 29,751 staff from 742 LHDs in 48 states to the 2021 Public Health Workforce Interests and Needs Survey, plus LHD characteristics and county demographics. Logistic regression assessed associations between key factors and staff-reported “knowledge of” and “confidence in addressing” structural racism, health equity, social determinants of equity (SDoE), social determinants of health (SDOH), and environmental justice, as well as belief and involvement in addressing racism through one’s work. Staff with a master’s degree or higher compared to others had greater odds of reporting confidence in addressing structural racism (adjusted odds ratio [AOR] = 1.23) and health equity (AOR = 1.56), agreeing that addressing racism should be a part of their work (AOR = 2.45) and being involved in such efforts (AOR = 1.57). Staff identifying as Black, compared to white, had greater odds of reporting confidence in addressing all concepts: structural racism (AOR = 1.98), health equity (AOR = 1.34), SDoE (AOR = 1.53), SDOH (AOR = 1.21), and environmental justice (AOR = 1.72) and agreeing that addressing racism should be a part of their work (AOR = 2.11). Patterns were similar among staff identifying as Hispanic/Latino and other persons of color. Black (AOR = 0.68) and Hispanic/Latino (AOR = 0.83) staff had lower odds, however, of reporting engagement in activities to address racism. Finally, competencies positively associated with nearly all outcomes included cross-sector collaboration, ability to incorporate health equity into programming, and policy advocacy. LHD workforce development should include training that involves explicitly naming structural racism’s effects and complementary skills, such as policy development and cross-sector partnership building. Further exploration is needed into how best to grow commitment among white staff and to support staff of color in health equity work.

## Introduction/Background

Public health departments are administrative units of local or state government and are responsible for protecting and promoting the health of their community [1]. At the local level, public health departments are typically organized to serve a U.S. county, but can serve multiple counties or a large city. Advancing health equity is at the core of the responsibility of these local health departments (LHDs) and is at the center of the Ten Essential Public Health Services framework, which describes public health activities that the LHD staff should perform in communities [2]. These activities include investigating and addressing health threats, preventing or minimizing adverse health effects from things like communicable disease, water or food contamination, and environmental hazards, coordinating emergency response, coordinating and collaborating with others in the community (such as health care institutions, community-based organizations) to identify and address sources of health problems, and provides support in maintaining up-to-date public health policies. The LHD staff carrying out these activities represent a range of professional disciplines and areas of expertise, such as epidemiologists, health educators, sanitarians, clinicians, and community health workers. Similarly, the “Health in All Policies” approach provides a framework to embed health equity considerations into LHD staff decision-making [3]. The COVID-19 pandemic raised awareness of existing health inequities and the LHD workforce’s important role in facilitating equitable access to health and social services and addressing social determinants of health. The ability of LHD staff to promote equity and address health disparities is shaped by financial, community, and organizational factors, including a health department’s rural versus urban location, existing partnerships, agency size, accreditation status, leadership commitment and support, types of staff, and types of services [4,5]. LHD workforce capacity for health equity work is also an important area needing attention as studies have found that the governmental public health workforce, of which LHD staff are a part, may lack the needed confidence and skills, such as the ability to engage populations that have been historically marginalized, and the commitment needed for this work [5–7].

Little research exists on health equity competencies for the public health workforce, including LHD staff. Existing studies tend to evaluate competencies using the Bay Area Regional Health Inequities Initiative’s self-assessment tool [8]. These studies suggest that commitment and support from agency leadership, explicitly naming health equity in agency strategies and goals, and providing resources for training are evidence of greater LHD commitment to health equity work and are consequential for addressing inequities [5,6]. A newly available tool is the “Racial Justice Competency Model for Public Health Professionals,” launched in 2022, which has 51 competency statements that address “introductory,” “intermediate,” and “leading” levels to provide guidance and support for development of racial justice training for public health professionals committed to achieving health equity through dismantling racism’s underlying causes [9]. Despite establishing competencies for promoting health equity and addressing the ways in which inequities are rooted in racism, most studies in this area have been descriptive and lack guidance for developing these competencies among staff. We know of no national or international studies that have identified facilitators for developing public health workforce competencies directly associated with achieving health equity or that demonstrate how LHDs operationalize a commitment to advancing health equity through their workforce [10].

Assessing public health workforce competencies associated with health equity has been difficult in part due to a lack of pertinent quantitative data. The de Beaumont Foundation’s 2021 Public Health Workforce Interests and Needs Survey (PH WINS), however, included new national survey items related to staff knowledge of and confidence in addressing five concepts: (1) health equity, (2) social determinants of equity (SDoE), (3) social determinants of health (SDOH), (4) structural racism, and (5) environmental justice [11,12]. Each of these concepts represents significant underlying factors perpetuating health inequities and, thus, are critical areas in which public health staff must feel equipped to act [7,13]. Recent research using these data showed that a majority of governmental public health staff reported awareness of the concepts of health equity, SDOH, and SDoE, but significantly fewer reported confidence in addressing these concepts through their work, particularly SdoE [7]. These findings highlight the need for further research into understanding factors supporting the governmental public health workforce to become more aware, confident, and ultimately effective, in their work to advance health equity. Beyond assessing confidence in addressing such underlying factors, it is also important to assess staff beliefs regarding their role in addressing racism, which further influences staff effectiveness [5,14].

To address these needs, our study explored the associations of multiple individual, community, and organizational characteristics with key competencies, beliefs, and activities reported by LHD staff that are important for advancing health equity. Findings can inform workforce development planning to ensure that this workforce can promote health equity in their communities.

## Methods

### Design and data set

For this cross-sectional study, we compiled a national data set including individual-level LHD staff characteristics and reported competencies, organizational-level LHD characteristics, and county-level demographics. Individual-level staff variables came from the 2021 PH WINS, a nationally representative survey of state and local health department staff in the U.S., administered by the de Beaumont Foundation and the Association of State and Territorial Health Officials. This survey, sent to 137,446 non-supervisors, supervisors, managers, and executives of state and local health departments, had a response rate of 35% [11,15]. Further details on survey methods can be found elsewhere [11]. LHD organizational information came from the 2019 National Profile of Local Health Departments Survey (“Profile”) (administered by the National Association of County and City Health Officials [NACCHO]) [16]. County-level demographics were retrieved from the 2020 Area Health Resource File (AHRF) and the 2020 Centers for Disease Control and Prevention/Agency for Toxic Substances and Disease Registry Social Vulnerability Index (SVI) data [17,18]. We linked PH WINS and Profile data using NACCHO identifiers and AHRF data via county-level Federal Information Processing System (FIPS) codes. We restricted our final sample to a total of 29,751 staff responding to PH WINS from 742 LHDs representing each of the 10 U.S. Health and Human Services Regions. This study was determined not to be human subjects research according to the University of Washington Institutional Review Board.

### Measures

#### Outcome variables

We explored 12 binary outcomes of interest. Ten were staff-reported “knowledge of” and “confidence in addressing” each of five concepts: structural racism, health equity, SDoE, SDOH, and environmental justice [12]. Likert scales measured knowledge (1 = No knowledge, 2 = Not much, 3 = A little, and 4 = A lot) and confidence (1 = Do not know concept, 2 = Not confident, 3 = A little confident, and 4 = Very confident). Definitions for each of these were provided to respondents (S1 Text); briefly, we define each as follows:

- **Structural Racism**: Dimensions of history and culture that provide enduring privileges associated with “whiteness” and disadvantages associated with color [19].
- **Health Equity**: All people have a just opportunity to live their healthiest lives (adapted from CityHealth [20].
- **SDoE**: Systems of power that determine distribution of resources and populations, including sexism, structural racism, nativism, and poverty [21].
- **SDOH**: The conditions of the places where people are born, live, work, play, and worship [22].
- **Environmental Justice**: Involvement of all people in the development, implementation, and enforcement of environmental laws and policies [23].

We transformed these 10 outcomes into binary variables—knowledge (0 = None/Not much/A little and 1 = A lot) and confidence (0 = Do not know/Not confident/A little confident and 1 = Very confident). The final two outcomes measured (1) staff’s agreement with whether “addressing racism as a public health crisis should be a part of my work” (yes or no) and (2) whether staff reported being “involved in efforts to address racism as a public health crisis” (0 = none and 1 = little/some/a lot). For the latter outcome, the difference between “a little” versus “some” involvement was vague, so we included both options with “a lot” to collectively represent “any involvement” in addressing racism as a public health crisis.

#### Independent variables

We selected independent variables of interest based on literature discussing key organizational and individual characteristics that can influence knowledge of health equity concepts and engagement in health equity work [5,24]. The two main documents that informed variable selection were the “Racial Justice Competency Model for Public Health Professionals” and the “Local Health Department Organizational Self-Assessment for Addressing Inequities” [8,9].

Individual-level indicators included *tenure* as a public health employee in their LHD (5 years or less versus more than 5 years), *racial and/or ethnic identity* (identifying as Black, Hispanic/Latinx or Other person of color [POC]) versus white), *highest degree* (bachelor’s or lower versus master’s or higher), and *primary program* categorizing staff into four areas: Maternal and Child Health (MCH), Environmental Health, COVID-19 compared to other (a composite of all other primary program areas). The four primary program categories that were specified had the largest representation in the sample. “Other POC” included Native Hawaiian/Pacific Islander, American Indian/Alaska Native, and Multi-race identities, combined to protect the anonymity of participants. While race and ethnicity are social constructs with no biological meaning, they can be important in efforts such as this to understand the influences of these identities on health equity competencies and work, and influences such as staffs’ varied backgrounds. Understanding the many identities and perspectives that staff bring to their work is necessary to address the ways *structural racism* (see S1 Text for full definition) operates to maintain a racial hierarchy [19,25]. We chose to include respondents’ self-reported racial and/or ethnic identity in our model due to the many ways that structural racism underlies inequities experienced by individuals who are not socially assigned “white” and how perspectives may differ among such individuals due to these experiences [26].

We also included the following binary skill measures important in developing health equity competencies, taken from the PH WINS survey (0 = No/Low proficiency and 1 = Proficient/Expert) [5,9,24,27]. Self-reported proficiencies for each skill were measured at the non-supervisor, supervisor, and executive levels separately. These responses were combined such that each skill variable included responses from staff at all levels of the LHD for that specific skill. Wording for each skill was slightly different depending on the level of the staff participant and as such, some skills have combined wording (e.g. “Assess/Advocate”). Detailed wording for each skill by staff level can be found in the S2 Text:

- Targeted communication (“Communication [Targeted]”)
- Persuasive communication (“Communication [Persuasive]”)
- Cross-sector partnership-building to address SDOH (“Cross-sector SDOH partnering”)
- Collaborate across agencies/public health systems (“Cross-agency collaboration”)
- Assess/Advocate for needed population health services (“Advocate for needed health services”)
- Identify/Engage community assets to improve community health (“Engage community assets”)
- Engage the community in program design and implementation (“Engage community [program implementation]”)
- Incorporate health equity and social justice concepts into programming (“Health equity programming”)
- Identify/Ensure use of appropriate sources of data (“Ensure appropriate use of data”)
- Identify and influence policies external to the organization affecting community health (“Identify/influence policies”)

Organizational indicators were whether staff worked in an LHD that was *clinician-led* and/or that was Public Health Accreditation Board (PHAB) *accredited*. *Clinician-led* was defined as an LHD with a lead executive with any of the following clinical degrees: Associate Degree in Nursing, Bachelor of Science in Nursing, Master of Science in Nursing, Doctorate of Nursing Practice, Doctor of Medicine or Doctor of Osteopathic Medicine [28–30].

#### Covariates

We accounted for additional county level characteristics that could influence individual staff capacity [31]. Rural/urban location was measured based on classifications of LHDs in the Profile survey [16]. The SVI represents a county’s relative socio-economic vulnerability based on five county-level measures (percentages): below 150% poverty level, unemployed, with no high school diploma, uninsured, and with a housing cost burden (households that spend 30% or more of annual income on housing costs [17]. We also accounted for health resources available by including *clinicians per capita* in the final model. We defined “clinicians” as including medical doctors, osteopathic doctors, and nurse practitioners.

We included additional county level characteristics to describe the overall sample: population size, median household income, % persons at or below the federal poverty level, % unemployed, and % 25 years or older with less than a high school diploma. However, these were not included in the final model due to measuring similar characteristics as the SVI.

### Analysis

We examined data for outliers and calculated descriptive statistics, including χ^2^ tests for bivariate analyses, evaluating significance at *P*<0.05. We used multivariable logistic regression analyses to assess odds ratios for each outcome of interest based on individual- and organizational-level predictors. We also conducted sensitivity analyses using multi-level modeling techniques to account for clustering within and between LHDs; however, clustering accounted for very little variance, so we do not report these results. We present the fully adjusted models below with all coefficients in their exponentiated form. All analyses were completed in STATA version 14 (Stata Corp, College Station, TX).

## Results

Most respondents identified as a woman, white and had a bachelor’s degree or less; nearly half were ages 31–50 years (Table 1). Most respondents also had worked in public health for more than 5 years, served in non-supervisor (Tier 1) roles, and worked in an urban and/or accredited LHD. Of those surveyed, 17% worked in MCH as their primary program area, 20% in COVID-19 response, and 9% in Environmental Health. The proportion of respondents who reported skill proficiencies ranged from 71% (targeted communication) to 30% (identify/influence policies affecting community health). Most staff reported having knowledge of the five health equity competency concepts we examined, with more having heard of health equity (69%) and SDOH (62%) than structural racism (47%), SDoE (48%), and environmental justice (40%). Proportionally fewer staff reported confidence in addressing each of these concepts, with the largest proportional difference among those reporting “a lot of confidence” addressing health equity (46%), structural racism (30%), and SDoE (32%). Most (70%) agreed that “Addressing racism as a public health crisis should be part of my work”; 66% reported involvement in efforts to do this work (Table 2).

**Table 1:** Characteristics of study population participating in PHWINS.

	Study population (n = 29751)
**Individual characteristics**	
**Gender, n (%)**	
Women	23518 (79)
Men	5355 (18)
Other*	488 (2)
**Age, n (%)**	
<31	3877 (13)
31–50	12995 (44)
50+	9936 (33)
**Race/ethnicity, n (%)**	
American Indian or Alaska Native	276 (1)
Asian	1810 (6)
Black or African American	4327 (15)
Hispanic or Latino	5749 (19)
Native Hawaiian or other Pacific Islander	95 (0.3)
White	15484 (52)
Two or more races	1238 (4)
**Supervisory status, n (%)**	
Nonsupervisor (Tier 1)	22316 (75)
Supervisor/Manager (Tier 2)	6700 (23)
Executive (Tier 3)	735 (2)
**Education level, n (%)**	
Bachelor’s degree or lower	20582 (69)
Master’s degree or higher	8732 (29)
**Tenure in public health practice, n (%)**	
0–5 years	10746 (36)
More than 5 years	17376 (58)
**Location, n (%)**	
Urban	24922 (84)
Rural	4829 (16)
**Primary program area, n (%)**	
Maternal and Child Health	4943 (17)
Environmental Health	2693 (9)
COVID-19	5854 (20)
Other	16240 (55)
**Organization characteristics**	
Lead executive is a clinician, n (%)	10866 (37)
Accredited, n (%)	17620 (59)
FTEs per 1,000 capita, mean (SD)	0.1 (0.4)
**Community characteristics, mean (SD)**	
Population, 1000’s	258 (642)
Median household income, $1000’s	58 (16)
Percent persons at or below federal poverty level	14 (4)
Percent unemployed	3 (1)
Percent 25 years+ with < high school diploma	8 (4)
Social Vulnerability Index (SVI)	0.5 (0.3)
Clinicians per capita	33 (25)
Percent Black	12 (15)
Percent American Indian/Alaska Native	1 (3)
Percent Hispanic/Latinx	8 (11)

*Other includes those identifying as non-binary.

**Table 2:** Distribution of staff reported proficiencies in select skills and outcomes from the 2021 PH WINS.

	Study population (n = 29751)
**Individual-level skills, n (%)**	
Targeted communication	21238 (71)
Persuasive communication	21978 (74)
Cross-sector SDOH partnering	13772 (46)
Cross-agency collaboration	14830 (50)
Advocate for needed health services	10697 (36)
Engage community assets	12074 (41)
Engage community in program implementation	11208 (38)
Health equity programming	15225 (51)
Ensure appropriate use of data	16570 (56)
Identify/influence policies	8856 (30)
**Endorsed outcomes, n (%)**	
Knowledge of structural racism	14045 (47)
Confidence in addressing structural racism	8902 (30)
Addressing racism as a public health crisis should be part of work	20843 (70)
Involved in efforts to address racism as a public health crisis	19492 (66)
Knowledge of health equity	20527 (69)
Confidence in addressing health equity	13581 (46)
Knowledge of SDOE	14266 (48)
Confidence in addressing SDOE	9537 (32)
Knowledge of SDOH	18164 (61)
Confidence in addressing SDOH	12563 (42)
Knowledge of environmental justice	11915 (40)
Confidence in addressing environmental justice	7764 (26)

PH WINS = Public Health Workforce Interests and Needs Survey; SDOH = Social determinants of health; SDoE = Social determinants of equity.

### Individual factors

Staff with a master’s degree or higher had higher odds, compared to those with less education, of reporting knowledge of all concepts of interest: structural racism (Adjusted Odds Ratio[AOR] = 1.94; 95% CI = 1.77–2.13), health equity (AOR = 2.69; 95% CI = 2.36–3.06), SDoE (AOR = 1.13; 95% CI = 1.04–1.24), SDOH (AOR = 3.00; 95% CI = 2.64–3.35), and environmental justice (AOR = 1.48; 95% CI = 1.35–1.62) (Tables 3–5). Those with more formal education also had higher odds of reporting confidence in addressing three of these areas: structural racism (AOR = 1.23; 95% CI = 1.12–1.36), health equity (AOR = 1.56; 95% CI = 1.41–1.72), and SDOH (AOR = 2.19; 95% CI = 1.98–2.42). Compared to those with a shorter tenure, staff with a longer tenure in public health (5+ years) had higher odds of reporting knowledge of health equity (AOR = 1.4; 95% CI = 1.26–1.57) and SDOH (AOR = 1.36; 95% CI = 1.23–1.5). However, longer-tenured staff had lower odds of reporting confidence in addressing structural racism (AOR = 0.77; 95% CI = 0.69–0.84) and SDoE (AOR = 0.89; 95% CI = 0.81–0.98) (Tables 3–5). Higher education and longer tenure were both positively associated with involvement in efforts to address racism as a public health crisis (AOR = 1.57; 95% CI = 1.40–1.76 & AOR = 1.14; 95% CI = 1.03–1.27, respectively); however, only those with higher education were also more likely to agree that such involvement should be part of their work (AOR = 2.45; 95% CI = 2.16–2.77) (Table 3).

**Table 3:** Factors associated with knowledge, confidence, public health work, and involvement in addressing structural racism.

	Knowledge of structural racism	Confidence in addressing structural racism	Addressing racism as a public health crisis should be part of work	Involved in efforts to address racism as a public health crisis
	Odds ratio (95% CI)	Odds ratio (95% CI)	Odds ratio (95% CI)	Odds ratio (95% CI)
**Individual characteristics**				
Tenure >5 years	0.97 (0.88, 1.06)	**0.78 (0.70, 0.86)**	0.90 (0.81, 1.01)	**1.14 (1.03, 1.27)**
Masters/PhD	**1.94 (1.77, 2.13)**	**1.23 (1.11, 1.36)**	**2.46 (2.17, 2.78)**	**1.57 (1.40, 1.76)**
Race/ethnicity				
Black	0.98 (0.85, 1.13)	**1.98 (1.70, 2.31)**	**2.11 (1.72, 2.59)**	**0.68 (0.58, 0.80)**
Hispanic/Latino	0.92 (0.81, 1.04)	**1.31 (1.15, 1.50)**	1.05 (0.91, 1.23)	**0.83 (0.72, 0.96)**
Other POC	0.98 (0.85, 1.13)	**1.23 (1.06, 1.43)**	0.96 (0.81, 1.15)	1.11 (0.92, 1.33)
Primary program area				
Maternal and Child Health	**1.32 (1.17, 1.49)**	1.06 (0.93, 1.20)	**1.43 (1.23, 1.66)**	**1.42 (1.23, 1.65)**
Environmental Health	**0.82 (0.69, 0.97)**	**0.81 (0.67, 0.98)**	**0.77 (0.64, 0.92)**	**0.73 (0.61, 0.88)**
COVID-19	**1.18 (1.06, 1.32)**	1.13 (1.00, 1.27)	**1.24 (1.08, 1.42)**	1.10 (0.97, 1.26)
**Individual-level skills**				
Targeted communication	0.90 (0.77, 1.04)	1.03 (0.86, 1.23)	0.71 (0.59, 0.85)	1.12 (0.95, 1.32)
Persuasive communication	1.03 (0.89, 1.19)	1.00 (0.84, 1.19)	0.93 (0.78, 1.11)	**0.84 (0.71, 0.99)**
Cross-sector SDOH partnering	**1.41 (1.26, 1.59)**	**1.54 (1.35, 1.76)**	**1.38 (1.20, 1.59)**	**1.34 (1.17, 1.53)**
Cross-agency collaboration	1.13 (1.00, 1.29)	1.12 (0.97, 1.31)	**1.22 (1.04, 1.43)**	**1.22 (1.05, 1.42)**
Advocate for needed health services	**1.16 (1.03, 1.59)**	**1.36 (1.19, 1.56)**	0.97 (0.83, 1.13)	**1.20 (1.03, 1.38)**
Engage community assets	0.96 (0.84, 1.09)	1.00 (0.87, 1.15)	**0.85 (0.73, 0.99)**	0.99 (0.85, 1.15)
Engage community in program implementation	1.13 (0.99, 1.28)	**1.24 (1.08, 1.43)**	1.17 (1.00, 1.38)	1.05 (0.90, 1.23)
Health equity programming	**1.52 (1.35, 1.70)**	**2.47 (2.16, 2.84)**	1.15 (1.00, 1.32)	**1.39 (1.21, 1.58)**
Ensure appropriate use of data	1.00 (0.88, 1.13)	0.94 (0.81, 1.09)	1.00 (0.86, 1.16)	0.88 (0.76, 1.02)
Identify/influence policies	1.04 (0.92, 1.17)	**1.26 (1.11, 1.43)**	**0.81 (0.69, 0.93)**	**0.74 (0.64, 0.86)**
**Organization characteristics**				
Clinician leader	**1.10 (1.01, 1.20)**	1.07 (0.97, 1.18)	1.06 (0.95, 1.18)	1.04 (0.94, 1.16)
Accredited	**0.87 (0.78, 0.97)**	0.97 (0.87, 1.09)	0.98 (0.86, 1.11)	0.88 (0.78, 1.00)
FTEs per 10,000 capita	1.00 (1.00, 1.00)	1.00 (1.00, 1.00)	1.00 (1.00, 1.00)	1.00 (1.00, 1.00)
**Community characteristics**				
Urban location	**1.46 (1.28, 1.67)**	**1.43 (1.23, 1.67)**	**1.37 (1.18, 1.59)**	1.15 (0.99, 1.33)
Social Vulnerability Index	**0.69 (0.54, 0.87)**	**1.45 (1.11, 1.89)**	**0.42 (0.32, 0.56)**	**0.71 (0.53, 0.93)**
Clinicians per capita	1.01 (1.01, 1.01)	1.01 (1.00, 1.01)	1.01 (1.00, 1.01)	1.01 (1.00, 1.01)
Percent Hispanic	1.00 (1.00, 1.01)	1.00 (1.00, 1.00)	1.01 (1.00, 1.01)	1.00 (0.99, 1.00)
Percent AIAN	1.00 (0.99, 1.01)	0.99 (0.98, 1.01)	1.00 (0.99, 1.02)	1.00 (0.99, 1.02)
Percent Black	0.99 (0.99, 1.00)	1.00 (0.99, 1.00)	0.99 (0.99, 1.00)	0.99 (0.99, 1.00)

POC = Person of color; SDOH = Social determinants of health; AIAN = American Indian/Alaskan Native.

**Table 4:** Factors associated with knowledge of and confidence in addressing health equity and social determinants of equity (SDoE).

	Knowledge of health equity	Confidence in addressing health equity	Knowledge of SDoE	Confidence in addressing SDoE
	Odds ratio (95% CI)	Odds ratio (95% CI)	Odds ratio (95% CI)	Odds ratio (95% CI)
**Individual characteristics**				
Tenure >5 years	**1.38 (1.23, 1.54)**	0.97 (0.88, 1.06)	1.07 (0.98, 1.17)	**0.89 (0.81, 0.98)**
Masters/PhD	**2.71 (2.38, 3.09)**	**1.56 (1.42, 1.72)**	**1.13 (1.04, 1.24)**	1.06 (0.96, 1.17)
Race/ethnicity				
Black	0.85 (0.71, 1.02)	**1.34 (1.15, 1.57)**	1.09 (0.95, 1.26)	**1.53 (1.32, 1.78)**
Hispanic/Latino	**0.78 (0.67, 0.91)**	0.99 (0.87, 1.13)	1.04 (0.92, 1.17)	**1.21 (1.06, 1.38)**
Other POC	**0.74 (0.62, 0.88)**	1.00 (0.86, 1.16)	1.05 (0.91, 1.20)	**1.26 (1.08, 1.46)**
Primary program area				
Maternal and Child Health	**1.18 (1.02, 1.37)**	1.14 (1.00, 1.28)	**1.31 (1.16, 1.47)**	**1.17 (1.03, 1.33)**
Environmental Health	**0.56 (0.47, 0.68)**	**0.52 (0.43, 0.62)**	**0.71 (0.60, 0.83)**	**0.64 (0.53, 0.77)**
COVID-19	**1.18 (1.02, 1.36)**	**1.18 (1.05, 1.33)**	1.11 (1.00, 1.24)	1.11 (0.99, 1.24)
**Individual-level skills**				
Targeted communication	0.97 (0.82, 1.15)	1.07 (0.92, 1.25)	1.04 (0.90, 1.20)	1.15 (0.96, 1.36)
Persuasive communication	1.08 (0.92, 1.28)	1.08 (0.93, 1.26)	1.11 (0.96, 1.28)	1.15 (0.97, 1.36)
Cross-sector SDOH partnering	**1.61 (1.40, 1.85)**	**1.61 (1.43, 1.82)**	**1.25 (1.11, 1.40)**	**1.53 (1.35, 1.74)**
Cross-agency collaboration	**1.65 (1.41, 1.93)**	**1.44 (1.26, 1.64)**	1.03 (0.91, 1.17)	1.06 (0.92, 1.23)
Advocate for needed health services	1.12 (0.95, 1.30)	**1.32 (1.16, 1.50)**	**1.19 (1.05, 1.34)**	**1.40 (1.24, 1.60)**
Engage community assets	1.16 (0.99, 1.36)	1.13 (0.99, 1.28)	**1.14 (1.01, 1.28)**	1.10 (0.96, 1.26)
Engage community in program implementation	0.96 (0.82, 1.14)	1.13 (0.99, 1.29)	1.13 (1.00, 1.29)	**1.26 (1.10, 1.45)**
Health equity programming	**1.19 (1.03, 1.37)**	**2.24 (2.00, 2.52)**	**1.51 (1.35, 1.69)**	**2.13 (1.87, 2.42)**
Ensure appropriate use of data	0.97 (0.84, 1.13)	1.01 (0.89, 1.15)	0.97 (0.86, 1.09)	1.04 (0.91, 1.20)
Identify/influence policies	**0.72 (0.62, 0.84)**	1.00 (0.88, 1.13)	1.00 (0.89, 1.12)	**1.21 (1.07, 1.37)**
**Organization characteristics**				
Clinician leader	1.10 (0.99, 1.23)	**1.16 (1.06, 1.28)**	**1.13 (1.03, 1.23)**	**1.11 (1.01, 1.22)**
Accredited	**1.25 (1.10, 1.43)**	1.05 (0.94, 1.17)	**1.16 (1.05, 1.29)**	**1.13 (1.01, 1.27)**
FTEs per 10,000 capita	1.00 (1.00, 1.00)	1.00 (1.00, 1.00)	1.00 (1.00, 1.00)	1.00 (1.00, 1.00)
**Community characteristics**				
Urban location	1.17 (1.00, 1.37)	**1.16 (1.01, 1.33)**	**1.20 (1.05, 1.37)**	**1.24 (1.07, 1.43)**
Social Vulnerability Index	**0.45 (0.34, 0.61)**	1.10 (0.86, 1.42)	0.95 (0.75, 1.20)	**1.59 (1.23, 2.05)**
Clinicians per capita	1.00 (1.00, 1.01)	1.00 (1.00, 1.00)	1.00 (1.00, 1.00)	1.00 (1.00, 1.00)
Percent Hispanic	1.00 (1.00, 1.00)	1.00 (1.00, 1.00)	1.00 (0.99, 1.00)	1.00 (0.99, 1.00)
Percent AIAN	1.01 (0.99, 1.02)	0.99 (0.98, 1.00)	1.00 (0.99, 1.01)	0.99 (0.98, 1.01)
Percent Black	0.99 (0.99, 1.00)	1.00 (0.99, 1.00)	0.99 (0.99, 1.00)	1.00 (0.99, 1.00)

POC = Person of color; SDOH = Social determinants of health; AIAN = American Indian/Alaskan Native.

**Table 5:** Factors associated with knowledge of and confidence in addressing social determinants of health (SDOH) and environmental justice.

	Knowledge of SDOH	Confidence in addressing SDOH	Knowledge of environmental justice	Confidence in addressing environmental justice
	Odds ratio (95% CI)	Odds ratio (95% CI)	Odds ratio (95% CI)	Odds ratio (95% CI)
**Individual characteristics**				
Tenure >5 years	**1.34 (1.21, 1.49)**	1.02 (0.92, 1.12)	0.99 (0.91, 1.09)	0.81 (0.74, 0.90)
Masters/PhD	**3.00 (2.66, 3.37)**	**2.19 (1.98, 2.42)**	**1.48 (1.35, 1.62)**	1.01 (0.91, 1.11)
Race/ethnicity				
Black	0.84 (0.71, 1.00)	**1.21 (1.04, 1.42)**	1.11 (0.97, 1.28)	**1.72 (1.48, 2.00)**
Hispanic/Latino	**0.71 (0.61, 0.82)**	0.91 (0.80, 1.04)	1.10 (0.97, 1.24)	**1.30 (1.13, 1.48)**
Other POC	**0.73 (0.61, 0.86)**	0.93 (0.79, 1.08)	**1.22 (1.06, 1.40)**	**1.22 (1.05, 1.43)**
Primary program area				
Maternal Child Health	**1.18 (1.03, 1.36)**	1.06 (0.94, 1.21)	**1.24 (1.10, 1.39)**	1.09 (0.95, 1.24)
Environmental Health	**0.48 (0.40, 0.57)**	**0.47 (0.39, 0.56)**	**1.89 (1.60, 2.23)**	**1.85 (1.54, 2.21)**
COVID-19	**1.29 (1.13, 1.47)**	**1.30 (1.15, 1.46)**	**1.30 (1.17, 1.45)**	**1.25 (1.11, 1.40)**
**Individual-level skills**				
Targeted communication	0.98 (0.84, 1.16)	1.05 (0.89, 1.23)	**0.85 (0.74, 0.99)**	1.06 (0.88, 1.28)
Persuasive communication	0.94 (0.80, 1.10)	0.96 (0.82, 1.12)	0.98 (0.85, 1.14)	1.16 (0.97, 1.40)
Cross-sector SDOH partnering	**1.76 (1.54, 2.00)**	**2.04 (1.81, 2.30)**	**1.29 (1.13, 1.43)**	**1.30 (1.14, 1.49)**
Cross-agency collaboration	**1.60 (1.38, 1.85)**	**1.32 (1.15, 1.51)**	1.06 (0.93, 1.21)	1.08 (0.92, 1.26)
Advocate for needed health services	1.12 (0.97, 1.30)	**1.45 (1.28, 1.65)**	**1.26 (1.11, 1.42)**	**1.41 (1.23, 1.61)**
Engage community assets	**1.16 (1.01, 1.34)**	1.09 (0.96, 1.24)	0.95 (0.84, 1.08)	1.06 (0.92, 1.22)
Engage community in program implementation	0.99 (0.85, 1.16)	**1.18 (1.03, 1.35)**	**1.23 (1.08, 1.40)**	**1.22 (1.05, 1.41)**
Health equity programming	1.03 (0.90, 1.17)	**1.73 (1.53, 1.95)**	**1.42 (1.26, 1.59)**	**2.04 (1.77, 2.34)**
Ensure appropriate use of data	1.10 (0.96, 1.27)	1.10 (0.97, 1.26)	1.09 (0.96, 1.23)	1.06 (0.92, 1.24)
Identify/influence policies	**0.83 (0.72, 0.96)**	1.03 (0.91, 1.16)	**1.15 (1.03, 1.29)**	**1.30 (1.14, 1.47)**
**Organization characteristics**				
Clinician leader	1.01 (0.91, 1.12)	1.06 (0.96, 1.16)	1.03 (0.94, 1.12)	1.08 (0.98, 1.20)
Accredited	**1.17 (1.03, 1.32)**	1.03 (0.92, 1.15)	1.06 (0.95, 1.17)	1.13 (1.00, 1.27)
FTEs per 10,000 capita	1.00 (1.00, 1.00)	1.00 (1.00, 1.00)	1.00 (1.00, 1.00)	1.00 (1.00, 1.00)
**Community characteristics**				
Urban classification	1.14 (0.98, 1.32)	**1.23 (1.06, 1.41)**	**1.20 (1.05, 1.37)**	1.16 (1.00, 1.36)
Economic Vulnerability Index	**0.49 (0.37, 0.64)**	1.19 (0.92, 1.54)	0.96 (0.76, 1.22)	**1.84 (1.41, 2.41)**
Providers per capita	1.01 (1.00, 1.01)	1.00 (1.00, 1.01)	1.00 (1.00, 1.01)	1.00 (1.00, 1.01)
Percent Hispanic	1.00 (1.00, 1.00)	1.00 (0.99, 1.00)	1.00 (1.00, 1.01)	1.00 (1.00, 1.00)
Percent AIAN	1.01 (0.99, 1.02)	0.99 (0.98, 1.01)	1.00 (0.99, 1.02)	0.99 (0.98, 1.01)
Percent Black	**0.99 (0.98, 0.99)**	0.99 (0.99, 1.00)	1.00 (0.99, 1.00)	1.00 (1.00, 1.01)

POC = Person of color; SDOH = Social Determinants of health; AIAN = American Indian/Alaskan Native.

Overall, staff who identified as Black/African-American, compared to those who identified as white, had higher odds of reporting confidence in addressing all concepts: structural racism (AOR = 1.98; 95% CI = 1.70–2.31), health equity (AOR = 1.34; 95% CI = 1.15–1.57), SDoE (AOR = 1.53; 95% CI = 1.32–1.78), SDOH (AOR = 1.21; 95% CI = 1.04–1.42), and environmental justice (AOR = 1.72; 95% CI = 1.48–2.00) (Tables 3–5). Compared to white staff, Black/African-American staff had 2.11 times greater odds of agreeing that “addressing racism as a public health crisis should be a part of my job” (95% CI = 1.72–2.59), but 0.68 lower odds of reporting involvement in such efforts (95% CI = 0.58–0.80) (Table 2). Similarly, the odds of staff who identified as Hispanic/Latino or Other POC of reporting confidence in addressing structural racism was 1.31 and 1.23 times that of white staff (95% CI = 1.15–1.50 & 1.06–1.43, respectively), while Hispanic/Latino staff had lower odds of reporting involvement in efforts to address racism (AOR = 0.83; 95% CI = 0.72–0.96) (Table 3). Further, Hispanic/Latino staff and Other POC staff had 0.78 and 0.74 times the odds, respectively, of reporting knowledge of health equity (95% CI = 0.67–0.91 & 0.62–0.88) and 0.71 and 0.73 times the odds, respectively, of reporting knowledge of SDOH (95% CI = 0.61–0.82 & 0.61–0.86) compared to white staff. Similar to Black/African American staff, these two groups had greater odds of reporting.

Confidence in addressing SDoE (AOR = 1.21; 95% CI = 1.06–1.38 & AOR = 1.26; 95% CI = 1.08–1.46, respectively) and environmental justice (AOR = 1.3; 95% CI = 1.13–1.48 & AOR = 1.22; 95% CI = 1.05–1.43, respectively) compared to white staff (Tables 4 and 5). The odds of other POC staff of reporting knowledge of environmental justice was also 1.22 times that of white staff (95% CI = 1.06–1.40).

Staff reporting that their primary program area was MCH had higher odds than staff in other areas of reporting knowledge of structural racism (AOR = 1.32; 95% CI = 1.17–1.49), agreeing that addressing racism as a public health crisis should be part of their work (AOR = 1.43; 95% CI = 1.23–1.66), and being involved in efforts to address this area (AOR = 1.42; 95% CI = 1.23–1.65) (Table 3). They were also more likely than others to report knowledge of all other examined concepts: health equity (AOR = 1.18; 95% CI = 1.02–1.37), SDoE (AOR = 1.31; 95% CI = 1.16–1.47), SDOH (AOR = 1.18; 95% CI = 1.03–1.36), and environmental justice (AOR = 1.24; 95% CI = 1.1–1.39) (Tables 3 and 4). Those working in COVID-19 response, compared to staff in other areas, were similarly more likely to report knowledge of all concepts except SDoE (Tables 3–5) and had 1.24 times greater odds of agreeing that addressing racism as a public health crisis should be part of their work (95% CI = 1.08–1.42). COVID-19 response staff also had higher odds of reporting confidence in addressing health equity (AOR = 1.18; 95% CI = 1.05–1.33), SDOH (AOR = 1.30; 95% CI = 1.15–1.46) and environmental justice (AOR = 1.25; 95% CI = 1.11–1.40). Those who primarily worked in Environmental Health had greater odds than staff in other areas of reporting knowledge of and confidence in addressing environmental justice (Knowledge: AOR = 1.89; 95% CI = 1.60–2.23 and Confidence: AOR = 1.85; 95% CI = 1.54–2.21), but lower odds of reporting knowledge of and confidence in addressing the other examined concepts (Tables 3 and 4).

### Skills

Staff who reported proficiency in cross-sector collaboration to address SDOH (“cross-sector SDOH partnering”) had higher odds of reporting knowledge of and confidence in addressing structural racism (AOR = 1.41; 95% CI = 1.26–1.59 & AOR = 1.54; 95% CI = 1.35–1.76), health equity (AOR = 1.61; 95% CI = 1.40–1.85 & AOR = 1.61; 95% CI = 1.43–1.82), SDOH (AOR = 1.76; 95% CI = 1.54–2.00 & AOR = 2.04; 95% CI = 1.81–2.30), and environmental justice (AOR = 1.28; 95% CI = 1.13–1.43 & AOR = 1.30; 95% CI = 1.14–1.49) (Tables 3–5). Compared to those not proficient, those reporting proficiency in cross-sector SDOH partnering had 1.38 greater odds of agreeing that addressing racism as a public health crisis should be part of their work (95% CI = 1.20–1.59) and 1.34 greater odds of being involved in efforts to address this (95% CI = 1.17–1.53).

Those reporting proficiency in incorporating health equity into programs and services (“health equity programming”) similarly were more likely to report knowledge of and confidence in addressing most of these areas; notably, those proficient in this skill had over twice the odds of those not proficient to report confidence in addressing structural racism (2.47; 95% CI = 2.16–2.84), health equity (2.24; 95% CI = 2.00–2.52), and SDoE (2.13; 95% CI = 1.87–2.42) (Tables 3 and 4).

Finally, having skills in health equity programming, along with proficiency in cross-agency collaboration, was positively associated with involvement in addressing racism as a public health crisis (AOR = 1.39; 95% CI = 1.21–1.58 & AOR = 1.22; 95% CI = 1.05–1.42), and reported proficiency in the latter skill was also positively associated with agreement that such involvement should be part of their work (AOR = 1.22; 95% CI = 1.04–1.43) (Table 3). Those reporting proficiency in engaging the community in program implementation had 1.24 times the odds of reporting confidence in addressing structural racism (95% CI = 1.08–1.43). Similarly, those proficient in advocating for needed policies were more likely to report knowledge of (AOR = 1.16; 95% CI = 1.03–1.59) and confidence in addressing structural racism (AOR = 1.36; 95% CI = 1.19–1.56) and had 1.20 times greater odds of reporting involvement in work to address racism as a public health crisis (95% CI = 1.03–1.38) (Table 3). In contrast, while the odds of reporting confidence in addressing racism for those reporting proficiency in identifying and influencing policies important for community health was 1.26 times that of those without this proficiency (95% CI = 1.11–1.43), the odds of agreeing that racism as a public health crisis should be part of their work and reporting involvement in such work for those with this policy proficiency was only 0.81 times and 0.74 times that of those without this proficiency (95% CI = 0.69–0.93 & 95% CI = 0.64–0.86, respectively).

### Organizational and community factors

The odds of staff in a clinician-led LHD of reporting knowledge of structural racism and SDoE were 1.10 and 1.13 times that of staff in non-clinician led LHDs (95% CI = 1.01–1.20 & 95% CI = 1.03–1.23, respectively) (Tables 3 and 4). Clinician-led LHD staff also had higher odds of reporting confidence in addressing health equity (AOR = 1.16; 95% CI = 1.06–1.28) and SDoE (AOR = 1.11; 95% CI = 1.01–1.22). Knowledge of and confidence in addressing the latter area was also higher among staff in an accredited LHD versus non-accredited (AOR = 1.16; 95% CI = 1.05–1.29 & AOR = 1.13; 95% CI = 1.01–1.27). The odds of accredited LHD staff reporting knowledge of health equity and SDOH were 1.25 and 1.17 times that of non-accredited staff (95% CI = 1.10–1.43 & 1.03–1.32) (Tables 3 and 4). However, this staff group was 0.87 times less likely than staff in non-accredited LHDs to report knowledge of structural racism (95% CI 0.78–0.97) (Table 3). Urban staff versus rural staff had higher odds of reporting knowledge of structural racism (AOR = 1.46; 95% CI = 1.28–1.67), health equity (AOR = 1.17; 95% CI = 1.00–1.37), SDoE (AOR = 1.20; 95% CI = 1.05–1.37) and environmental justice (AOR = 1.20; 95% CI = 1.05–1.37) (Tables 3–5). Urban staff were also more likely to report confidence in addressing four areas: structural racism (AOR = 1.43; 95% CI = 1.23–1.67), health equity (AOR = 1.16; 95% CI = 1.06–1.28), SDoE (AOR = 1.24; 95% CI = 1.07–1.43), and SDOH (AOR = 1.23; 95% CI = 1.06–1.41). Urban staff were 1.37 times more likely than rural staff to agree that addressing racism as a public health crisis should be a part of their work (95% CI = 1.18–1.59) (Table 3).

## Discussion

Overall, we found that higher education, involvement in MCH programs, and proficiencies in cross-sectoral collaboration and health equity programming were associated with greater knowledge of and confidence in addressing most or all health equity concepts examined, as were working in LHDs with clinician leaders and/or being located in urban areas. Longer tenure in public health or working primarily in environmental health were factors associated with less confidence in addressing most concepts. Individual and organizational factors that predicted concept knowledge and confidence were also frequently predictive of having the belief that addressing racism as a public health crisis should be a part of staff’s daily work and being involved in such work. These positive associations were also seen among Black or Hispanic staff, compared to white staff; however, Black and Hispanic staff were notably less likely than white staff to report being involved in work to address racism as a public health crisis.

We found that while having more education may be positively associated with competencies important for advancing health equity, longer tenure in public health is not consistently (and at times, is negatively) predictive. This suggests that staff with more formal education may have had more exposure to tools for addressing issues such as structural racism or health equity [32–34]. We do not know if these staff were exposed through formal or other educational experiences, but other research suggests that the quality of formal education in this area may be mixed, particularly with respect to content focused on racism. A study by Chandler et al. demonstrated that public health schools have “little pedagogical guidance” to inform their approaches to teaching students about health equity and racism [35]. This may be reflected in the magnitude of differences in odds ratios—those with higher education, compared to less, had 2.7 and 3 times the odds of reporting knowledge of health equity and SDOH, compared to an AOR of 1.5 with respect to knowledge of structural racism. Regarding tenure, five or more years’ tenure predicted lower confidence in concepts newer to some, such as structural racism and SDoE and lower odds of agreeing that addressing racism as a public health crisis should be a part of one’s work. This may reflect a lack of opportunity to grow in such confidence or potentially a lack of knowledge with where to start in addressing structural racism. As our sample was majority white-identifying, this could also reflect a pattern of thinking that racism is not a pressing problem as this group’s social privilege may make them less directly aware of its negative effects. This speaks to a need for better access to on-the-job training that involves application of learning and explicit naming of the effects of structural racism, as well as dedicated funding and leadership support, as described in recent research [7,36]. The “Racial Justice Competency Model for Public Health Professionals” is an example of a resource that offers helpful guidance for such training [9]. This set of competencies, developed through an iterative process with input from a diverse group of experts and aligned with the domains of the 10 Essential Public Health Services, provides a toolkit along with additional supports which can be used to identify areas where individual or organizational training is needed as well as develop training targeted to those areas.

Proficiency in skills related to health equity programming, cross-sector collaboration, advocating for needed policies, and community engagement stood out as being positively associated with knowledge of and confidence in addressing most concepts. This highlights the importance of including these skills in health equity-oriented trainings, thus, providing an overall “package” of skills for ensuring staff have needed competencies to effectively engage in health equity work. Our findings suggest that training in these skills needs to involve imparting a strong foundation of knowledge as well as application to increase confidence, regardless of tenure in public health. These associations also demonstrate the importance of public health staff working closely in and with the communities they serve—as those that indicated greater proficiency in engaging communities and building partnerships are also those who were more confident in addressing issues contributing to inequities such as structural racism. Certain skills overall did not show a relationship to knowledge and/or confidence, including those related to influencing policies for community health, data use, and communication; however, some of these skills would seem an important part of advancing health equity as previous research has found a relationship between skills related to data use and support for addressing SDOH [13].

There is a strong national interest in increasing workforce diversity as part of advancing health equity [37]. Our findings provide further support for such work as they suggest that a more diverse workforce may be associated with more staff with confidence in addressing issues such as structural racism and SDoE. This aligns with research on the overall PH WINS sample (inclusive of state health department staff), where proportionally more staff of color, compared to white staff, reported confidence in addressing health equity, SDOH, and SDoE [7]. However, our findings should not be used to justify increasing diversity in the workforce for the purposes of extracting these skills, which all staff need to cultivate; instead, the overall goal is to build a more inclusive and equitable workplace, as well as increase the public health workforce’s ability to serve diverse populations [38,39].

Staff of color were generally less likely to report being involved in efforts to address racism, despite being more likely to report that they agree it should be part of their work. The reason for this may reflect a difference between staff of color versus white in interpretation of what this effort should entail. Perhaps because staff of color were more likely to be serving in non-supervisor (e.g., administrative) roles and/or positions with less decision-making power, they felt less direct involvement in their LHD’s programmatic efforts to address inequities (see S1 Table). If so, they may perceive themselves as less involved or empowered to engage in activities directly related to addressing racism. Anti-racism work is often placed on top of existing responsibilities with research suggesting that people of color receive fewer resources than white staff to do DEI work. Adding more work that is challenging and requires significant mental and emotional energy while not reallocating other responsibilities hinders anti-racism work [40–42]. The racial differences found in this study may therefore reflect a lack of support for staff of color to do this work (e.g., failing to create accommodations, provide compensation, and endow the role with necessary authority) [43–45]. It may also reflect lack of opportunity or the reality that, in experiencing racism daily, staff of color may not wish to take on undue additional hardship [46,47].

At an organizational level, staff at clinician-led health departments were consistently more likely to report knowledge of the concepts examined, including structural racism, and to report confidence in addressing health equity and SDoE. Previous research has found that when clinicians lead LHDs, community health disparities are more significantly reduced, than when non-clinicians lead LHDs [28,48]. Our findings further reinforce the value of having a clinician in leadership. Staff at urban LHDs were also more likely to report having knowledge of and confidence in addressing each examined concept compared to rural peers. This too aligns with previous research exploring training needs among the rural public health workforce [49]. However, findings were more mixed for staff at accredited health departments. Accreditation was positively associated with knowledge of health equity, SDoE and SDOH, but negatively associated with knowledge of structural racism. Until very recently, accreditation standards did not explicitly require incorporation of policies or procedures showing evidence of the LHD’s commitment to address racism [50]. Further exploration of the impact of these newer accreditation standards is needed.

Given racism’s role in health inequities and the public health workforce’s role in advancing equity, staff’s belief that addressing racism should be a part of their work is critical [7]. Our findings suggest important modifiable factors that are positively associated with such beliefs and with active involvement in work to address racism. These factors include supporting higher education and/or continuing education, assuring a more diverse workforce, and growing proficiencies in collaborating across the public health system, cross-sector partnerships to address SDOH, advocating for needed policies, and incorporating health equity and social justice into programming. Addressing these factors, particularly in LHDs, can reinforce the impact of workforce development activities and guide the advancement of educational curricula for trainings that support a belief among public health staff, particularly white staff, that addressing racism should be part of every role [35,38]. In addition, work is needed to bolster diversity of the educational pathway from academic institutions to the workforce [51]. Finally, organizational work and resources are needed to ensure policies are equity-oriented and teams have shared language and knowledge in the context of diverse identities as well as a safe space for honest dialogue surrounding health equity concepts to support a solid foundation for doing this work [52].

Study limitations include the cross-sectional nature of the data, which prevents causal attributions. PH WINS, like all survey data, is subject to self-report bias. Because we did not use the weighting included with the PH WINS 2021 survey, our ability to generalize may be limited, but this strategy allowed us to have a larger sample size and include small, rural LHDs. In addition, our sensitivity analysis, using multi-level modeling techniques to account for clustering within and between LHDs, showed little difference in the results. While the sample was predominantly white, we were able to account for this in controlling for race and ethnicity in our regression analysis. We also acknowledge our own biases as authors who mostly identify as white (PK, BB, DP, and KS), limiting our team’s ability to interpret associations found between staff of color and certain competencies. For this reason, we were intentional in ensuring we had a variety of perspectives on our team, including team members who identified as a person of color as well as team members with expertise in public health practice (DZ, PK, BB, SS), public health workforce research (PK, BB, KS), and health equity capacity building (DZ, DP, PK).

## Conclusion

Our findings provide a deeper and more nuanced understanding of both individual- and organizational-level factors positively associated with a public health workforce equipped to advance health equity. This study’s implications support more targeted workforce development, including designing training to educate staff about health equity and structural racism. This training should include application of concepts and skill development in identifying, influencing, and implementing policies, community engagement. and cross-sector partnership building. Future research is needed to explore how to effectively support white staff to understand their role, as public health practitioners, in addressing racism, how to support staff of color in health equity work, and the types of work public health staff identify as being important for advancing health equity, the latter of which is a qualitative study currently being conducted by this research team. A diverse public health workforce that both values and is competent in addressing the root causes of inequities is essential in supporting equitable community health outcomes.

## Supporting information

S1 Text Health equity concept definitions. This table lists the definitions of the health equity concepts provided to PH WINS survey participants.
(DOCX)

S2 Text Detailed wording for skills included in model. This provides the full wording, by supervisory level, for the skills included in the model.
(DOCX)

S1 Table Supervisory level by race/ethnicity. Proportions of staff in each supervisory category– non-supervisor, supervisor, and manager/executive—by racial/ethnic identity.
(DOCX)
