# Supplementary material for: Individual and organizational factors associated with public health workforce competencies to advance health equity
Source: PLOS Glob Public Health. 2025 Jan 9;5(1):e0004068. doi: 10.1371/journal.pgph.0004068 (PMC11717272; doi:10.1371/journal.pgph.0004068)
Supplement: S1 Text — This table lists the definitions of the health equity concepts provided to PH WINS survey participants. (DOCX) [file pgph.0004068.s001.docx]

**Health Equity Competency Definitions**

**Health equity:** Health equity means all people, regardless of who they are, where they came from, how they identify, where they live, or the color of their skin, have a fair and just opportunity to live their healthiest possible lives - in body, mind, and community. Achieving health equity requires removing social, economic, contextual, and systemic barriers to health, and a continuous and explicit commitment to prioritize those affected by historical disadvantages. (CityHealth)

**Racism as a Public Health Crisis:** States, cities, and counties have increasingly declared racism to be a public health crisis or emergency. These declarations are driven by a recognition that systemic, institutional, and other forms of racism drive disparities across employment, housing, education, the justice system, healthcare, and other determinants of health. The declarations also reflect a growing acknowledgment that state and local governments must anchor efforts to eradicate the impacts of racism in order to truly achieve the conditions that create optimal health for all. (Network for Public Health Law)

**Social Determinants of Equity:** The social determinants of equity are systems of power like racism, sexism, heterosexism, ableism, and economic systems like capitalism. The social determinants of equity determine the range of contexts available and who is found in which context. They govern the distribution of resources and populations through decision-making structures, policies, practices, norms, and values, and too often operate as social determinants of in-equity by differentially distributing resources and populations (Jones, 2014)

**Structural Racism:** A system in which public policies, institutional practices, cultural representations, and other norms work in various, often reinforcing ways to perpetuate racial group inequity. It identifies dimensions of our history and culture that have allowed privileges associated with “whiteness” and disadvantages associated with “color” to endure and adapt over time. (Aspen Institute)

**Environmental Justice:** Environmental justice is the fair treatment and meaningful involvement of all people regardless of race, color, national origin, or income, with respect to the development, implementation, and enforcement of environmental laws, regulations, and policies. (US EPA)
