# Supplementary material for: Individual and organizational factors associated with public health workforce competencies to advance health equity
Source: PLOS Glob Public Health. 2025 Jan 9;5(1):e0004068. doi: 10.1371/journal.pgph.0004068 (PMC11717272; doi:10.1371/journal.pgph.0004068)
Supplement: S2 Text — This provides the full wording, by supervisory level, for the skills included in the model. (DOCX) [file pgph.0004068.s002.docx]

**S2. Skills included in model – Detailed wording by supervisory level**

| **Skill** | **Non-supervisors** | **Supervisors/Managers** | **Executives** |
| --- | --- | --- | --- |
| **Targeted Communication** | Effectively target communications to different audiences (e.g., the public, community organizations, external partners, the scientific community, etc.) | Communicate in a way that different audiences (e.g., the public, community organizations, external partners, the scientific community, etc.) can understand | Communicate in a way that different audiences (e.g., the public, community organizations, external partners, the scientific community, etc.) can understand |
| **Persuasive Communication** | Communicate in a way that persuades others to act | Communicate in a way that persuades others to act | Communicate in a way that persuades others to act |
| **Identify/Ensure use of appropriate sources of data** | Identify appropriate sources of data and information to assess the health of a community | Identify appropriate sources of data and information to assess the health of a community | Ensure the use of appropriate sources of data and information to assess the health of a community |
| **Incorporate health equity and social justice concepts into programming** | Support inclusion of health equity and social justice principles into planning for program and service delivery (e.g., include health equity in a strategic plan, promote health-in-all-policies, engage marginalized and under-resourced communities in decision making) | Incorporate health equity and social justice principles into planning for programs and services (e.g., include health equity in a strategic plan, promote health-in-all-policies, engage marginalized and under-resourced communities in decision making) | Incorporate health equity and social justice principles into planning across the agency (e.g., include health equity in a strategic plan, promote health-in-all-policies, engage marginalized and under-resourced communities in decision making) |
| **Identify/Engage community assets to improve community health** | Engage community assets and resources (e.g., Boys & Girls Clubs, public libraries, hospitals, faith-based organizations, academic institutions, federal grants,  fellowship programs) to improve health in a community | Identify and engage assets and resources (e.g., Boys & Girls Clubs, public libraries, hospitals, faith-based organizations, academic institutions, federal grants,  fellowship programs) that can be used to improve health in a community | Negotiate with multiple partners for the use of assets and resources (e.g., Boys & Girls Clubs, public libraries, hospitals, faith-based organizations, academic institutions, federal  grants, fellowship programs) to improve health in a community |
| **Cross-sector partnership-building to address SDOH** | Describe how social determinants of health impact the health of individuals, families, and the overall community | Build cross-sector partnerships (e.g., agencies or organizations supporting transportation, housing, education, and law enforcement) to address social determinants of health | Influence policies external to the organization that address social determinants of health (e.g., zoning, transportation routes, etc.) |
| **Collaborate across agencies/public health systems** | Collaborate with public health personnel across the agency to improve the health of the community | Engage in collaborations within the public health system, including traditional and non-traditional partners, to improve the health of a community. | Build collaborations within the public health system among traditional and non-traditional partners to improve the health of a community |
| **Assess/Advocate for needed population health services** | Describe your role in improving the health of the community served by the agency | Assess how agency policies, programs, and services advance population health | Advocate for needed population health services and programs |
| **Engage the community in program design and implementation** | Describe the importance of engaging community members in the design and implementation of programs to improve health in a community | Engage community members in the design and implementation of programs to improve health in a community | Ensure community member engagement in the design and implementation of programs to improve health in a community |
| **Identify and influence policies external to the organization affecting community health** | Collect and summarize information to inform the development of policies external to the organization that affect the health of the community (e.g., transportation routes, earned sick leave, tobacco 21, affordable housing/inclusionary zoning, complete streets, healthy food procurement) | Identify and assess options for policies external to the organization that affect the health of the community (e.g., transportation routes, earned sick leave, tobacco 21, affordable housing/inclusionary zoning, complete streets, healthy food procurement) | Prioritize and influence policies external to the organization that affect the health of the community (e.g., transportation routes, earned sick leave, tobacco 21, affordable housing/inclusionary zoning, |
