# Supplementary material for: Individual and organizational factors associated with public health workforce competencies to advance health equity
Source: PLOS Glob Public Health. 2025 Jan 9;5(1):e0004068. doi: 10.1371/journal.pgph.0004068 (PMC11717272; doi:10.1371/journal.pgph.0004068)
Supplement: S1 Table — Proportions of staff in each supervisory category– non-supervisor, supervisor, and manager/executive—by racial/ethnic identity. (DOCX) [file pgph.0004068.s003.docx]

| **S3 Appendix. Race/Ethnicity, Primary Program, and Individual-Level Skills of Study Population by Supervisory Status** | | | | |
| --- | --- | --- | --- | --- |
|  | **Tier 1**  **(n=22316)** | **Tier 2**  **(n=6700)** | **Tier 3**  **(n=735)** | ***p value*** |
| **Race/ethnicity, n (%)** |  |  |  | <0.001 |
| Black or African American | 3300 (15) | 952 (14) | 75 (10) |  |
| Hispanic or Latino | 4544 (20) | 1126 (17) | 79 (11) |  |
| Other POC | 2602 (12) | 748 (11) | 69 (9) |  |
| White | 11268 (50) | 3721 (56) | 495 (67) |  |
| **Primary program area, n (%)** |  |  |  | <0.001 |
| Maternal Child Health | 3841 (17) | 1076 (16) | 26 (4) |  |
| Environmental Health | 2035 (9) | 618 (9) | 40 (5) |  |
| COVID-19 | 4282 (19) | 1335 (20) | 237 (32) |  |
| Other | 12144 (54) | 3664 (55) | 432 (59) |  |
| **Individual-level skills, n (%)** |  |  |  |  |
| Targeted communication | 14976 (67) | 5608 (84) | 654 (89) | <0.001 |
| Persuasive communication | 15898 (71) | 5445 (81) | 635 (86) | <0.001 |
| Cross-sector SDOH partnering | 10369 (46) | 3026 (45) | 377 (51) | <0.001 |
| Cross-agency collaboration | 10936 (49) | 3351 (50) | 543 (74) | <0.001 |
| Advocate for needed health services | 7583 (34) | 2621 (39) | 493 (67) | <0.001 |
| Engage community assets | 8730 (39) | 2871 (43) | 473 (64) | <0.001 |
| Engage community in program implementation | 8152 (37) | 2608 (39) | 448 (61) | <0.001 |
| Health equity programming | 11182 (50) | 3575 (53) | 468 (64) | <0.001 |
| Ensure appropriate use of data | 11817 (53) | 4165 (62) | 588 (80) | <0.001 |
| Identify/influence policies | 6430 (29) | 2061 (31) | 365 (50) | <0.001 |
| POC=Person of color; SDOH=Social determinants of health | | | | |
